# Cellular calcium homeostasis and regulation of its dynamic perturbation

**DOI:** 10.1017/qpb.2025.2

**Published:** 2025-02-14

**Authors:** Colin Brownlee, Glen L. Wheeler

**Affiliations:** 1 Marine Biological Association, The Laboratory, Citadel Hill, Plymouth, UK; 2 School of Ocean and Earth Sciences, University of Southampton, Southampton, UK

**Keywords:** calcium, channel, endomembrane, homeostasis, plant

## Abstract

Calcium ions (Ca^2+^) play pivotal roles in a host of cellular signalling processes. The requirement to maintain resting cytosolic Ca^2+^ levels in the 100–200 nM range provides a baseline for dynamic excursions from resting levels that determine the nature of many physiological responses to external stimuli and developmental processes. This review provides an overview of the key components of the Ca^2+^ homeostatic machinery, including known channel-mediated Ca^2+^ entry pathways along with transporters that act to shape the cytosolic Ca^2+^ signature. The relative roles of the vacuole and endoplasmic reticulum as sources or sinks for cytosolic Ca^2+^ are considered, highlighting significant gaps in our understanding. The components contributing to mitochondrial, chloroplast and nuclear Ca^2+^ homeostasis and organellar Ca^2+^ signals are also considered. Taken together, a complex picture of the cellular Ca^2+^ homeostatic machinery emerges with some clear differences from mechanisms operating in many animal cells.

## Graphical abstract

**Figure:**
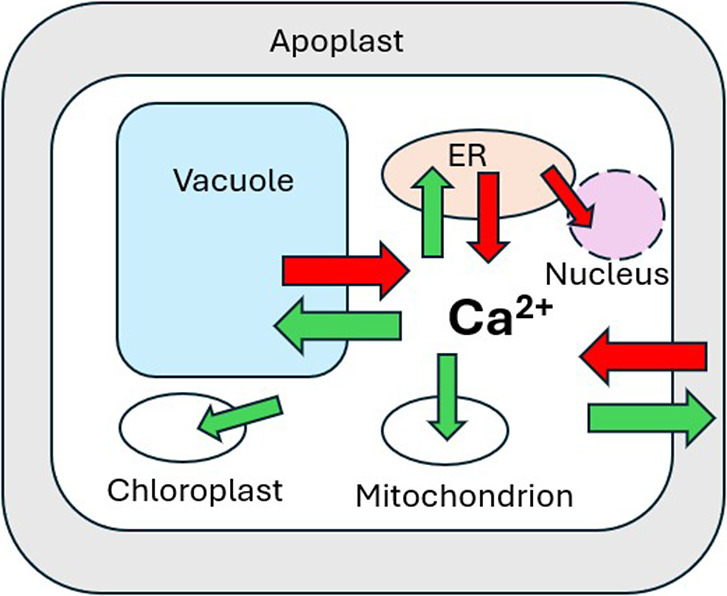

## 1. Introduction

Calcium ions (Ca^2+^) play multiple physiological and structural roles across prokaryote and eukaryote kingdoms. Eukaryotes maintain very low baseline cytosolic Ca^2+^ concentration [Ca^2+^_cyt_] of around 100–200 nM since Ca^2+^ is essentially toxic at concentrations even in the low micromolar range due, at least in part, to the ability to bind inorganic phosphate (Clapham, 2007). A typical plant cell is faced with an approximately 1,000-fold inwardly directed concentration gradient across the plasma membrane (PM), so maintaining very low [Ca^2+^_cyt_] requires highly efficient homeostatic mechanisms. In parallel to mechanisms for keeping [Ca^2+^_cyt_] low, cells have evolved mechanisms to allow controlled entry of Ca^2+^ into the cytosol, giving rise to tightly regulated Ca^2+^_cyt_ elevations that can act to relay signals from the cell surface to downstream response elements in the cell interior. In plants, patterns of Ca^2+^_cyt_ elevations vary considerably in response to different stimuli and include single transient elevations, lasting a few seconds, repeated oscillatory elevations over longer time periods and more prolonged elevations (Edel et al., 2017; Lenzoni et al., 2018). Moreover, Ca^2+^_cyt_ elevations may occur uniformly across the cell or maybe highly localized to a particular region (Brownlee & Wheeler, 2023). A further key feature of Ca^2+^ is its ability to bind reversibly to, and affect the activity of, a wide range of cellular regulatory proteins (Clapham, 2007; Edel et al., 2017) and different Ca^2+^_cyt_ elevation patterns represent signatures that can differentially activate a wide range of downstream elements (including calmodulin (CaM),calmodulin-like (CMLs), calcineurin B-like (CBLs), calcium-dependent protein kinases (CDPKs) and calcium/calmodulin kinases (CCamKs)) in stimulus- and cell-specific manners to bring about specific end responses (Demidchick et al., 2018; Edel et al., 2017; Lenzoni et al., 2018).

The generation of specific Ca^2+^_cyt_ signals involves the coordinate orchestration of channels, which allow passive movement of Ca^2+^ down its electrochemical potential gradient and active transporters that maintain resting [Ca^2+^_cyt_] and return Ca^2+^ to baseline levels, shaping the Ca^2+^_cyt_ signature. This review provides an assessment of the key components of the Ca^2+^ homeostatic and signalling machinery. We consider some of the most significant recent advances and key questions still to be addressed. These include: What determines the set points for Ca^2+^ homeostasis? What are the relative capacities and roles of the different cellular Ca^2+^ buffering compartments? To what extent do these compartments act as releasable Ca^2+^ stores and how does this vary with different signalling processes?

Addressing these questions requires quantitative assessments of the contribution of individual and coordinated cellular compartments in the regulation of Ca^2+^_cyt_. Until recently, measurement of concentrations and fluxes into and out of cellular compartments was primarily based on isolated organelles or microelectrode measurements of larger compartments, such as vacuoles. Recent years have witnessed a number of revolutionary advances, including improved electrophysiological approaches and the advent of targeted genetically encoded fluorescent Ca^2+^ reporters, that will provide new insights into the roles of different components of the Ca^2+^ homeostatic machinery and how its perturbation is finely controlled.

## 2. Components of the plant Ca^2+^ homeostat

### 2.1. Cytosolic buffers

**Figure 1.**
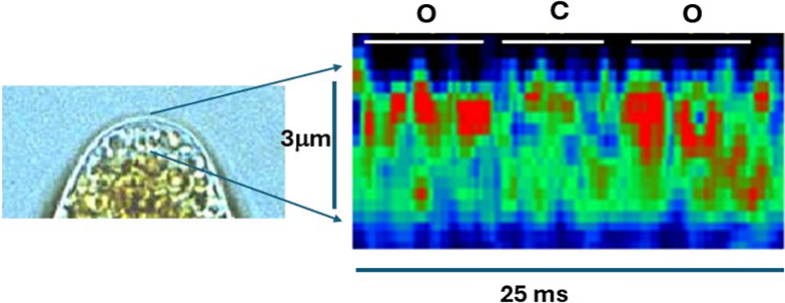
Highly localized Ca^2+^ elevations, visualized with the fluorescent Ca^2+^ indicator Calcium Green dextran, at the apex of a *Fucus serratus* rhizoid cell during the initiation of a Ca^2+^_cyt_ elevation in response to hypoosmotic treatment. Discrete Ca^2+^ elevations (red) are apparent and do not propagate >1 μm from the PM during putative channel opening (O) and disappear during channel closure (C). Adapted from Goddard et al. (2000).

A number of theoretical and experimental studies have shown that steady-state Ca^2+^_cyt_ levels in eukaryotic cells are determined primarily by the balance between influx and efflux mechanisms rather than passive cytosolic buffering (Eisner et al., 2023; Rios, 2010). However, cytosolic Ca^2+^ buffers do play an important role in modulating the rates of change of [Ca^2+^_cyt_] as well as the amplitude of Ca^2+^_cyt_ elevations in response to changes in membrane Ca^2+^ fluxes (Eisner et al., 2023). Consider a typical PM Ca^2+^ channel passing around 0.5 pA or ~10^6^ Ca^2+^ ions/s in a single cuboid plant cell of volume approximately 10^−14^ m^3^ and a typical cytosolic volume of 10% total cellular volume. A simple calculation reveals that in the absence of any cytosolic buffering a single Ca^2+^ channel with an open probability of 0.5 could potentially raise the [Ca^2+^_cyt_] at a rate of ~5 μM s^−1^. While there are no reliable estimates of Ca^2+^ channel density in the PM of plant or algal cells, single-channel patch clamp studies (e.g. Taylor et al., 1996; White et al., 1999) suggest a conservative estimate of 0.5 channels μm^−2^, which would equate to a total potential channel complement in the thousands. Clearly, if all Ca^2+^ channels in the PM open simultaneously, then without buffering or active mechanisms to remove Ca^2+^, [Ca^2+^_cyt_] could potentially elevate at a rate of several mM s^−1^ until equilibrium concentrations were achieved across the PM. A similar calculation based on a typical whole-cell plant Ca^2+^ current of around 100 pA gives a similar rate of [Ca^2+^_cyt_] increase in the absence of buffering. Since most whole-cell stimulus-induced Ca^2+^ transients do not reach peak values higher than the low μM, there must exist highly efficient mechanisms for Ca^2+^ buffering or removal. Passive Ca^2+^_cyt_ buffers include Ca^2+^-binding proteins, polyvalent inorganic and organic anions anionic lipid heads, and carboxyl residues (Demidchick et al., 2018; Eisner et al., 2023; Schwaller, 2010). While the buffering capacity of plant cytosol has not been precisely determined and will vary with cell type, in a typical animal cell, the presence of a range of Ca^2+^ buffers with Kd values slightly higher than resting [Ca^2+^_cyt_], suggests that passive buffering would become more effective as [Ca^2+^_cyt_] began to rise above resting levels (Schwaller, 2010). The affinities, concentrations, kinetics and mobilities of the Ca^2+^ buffers will subsequently determine the rate of [Ca^2+^_cyt_] elevation and its amplitude (Eisner et al., 2023; Neher, 1998; Wagner & Keizer, 1994), as well as the extent of Ca^2+^_cyt_ gradients resulting from localized PM Ca^2+^ fluxes in polarized plant cells, such as pollen tubes (e.g. Pierson et al., 1994). The ratio of bound/free Ca^2+^ in plant cytosol has been estimated to be >90% (Demidchick et al., 2018; Schonknecht & Bethmann, 1998) and as little as 1% of total Ca^2+^_cyt_ is considered to be free in a typical animal cell (Eisner et al., 2023), implying that an elevation of free [Ca^2+^_cyt_] from 200 nM to 2 μM would require an influx sufficient to increase total Ca^2+^_cyt_ by >20 μM. The impact of strong cytosolic Ca^2+^ buffering is also evident from a wide range of animal studies that have shown highly localized Ca^2+^ elevations (sparks) at sites of Ca^2+^ entry through channels that only propagate further through coordinated Ca^2+^-dependent Ca^2+^ release from intracellular stores (Cheung & Lederer, 2008). Non-equilibrium buffering of Ca^2+^ influx through a single channel is dependent on the rate of Ca^2+^ diffusion from the mouth of the channel and the probability that a Ca^2+^ ion will encounter the Ca^2+^ binding site of a buffer molecule, which is critically dependent on the buffer concentration (Stern, 1992). The extent of [Ca^2+^_cyt_] increase at the channel mouth will also depend on the rate of diffusion of Ca^2+^-buffer away from the channel as well as the buffer affinity, resulting in an exponential spatial [Ca^2+^_cyt_] decay profile (Stern, 1992). While there are no direct examples of such elemental Ca^2+^ elevations in vascular plants, these have been observed in rhizoid cells of the brown alga *Fucus serratus* in response to osmotically-induced channel activation on the PM and endomembranes (Goddard et al., 2000) (Figure 1).

### 2.2. *Ca*^***2+***^ ***entry pathways***

#### 2.2.1. Apoplast-plasma membrane

**Figure 2.**
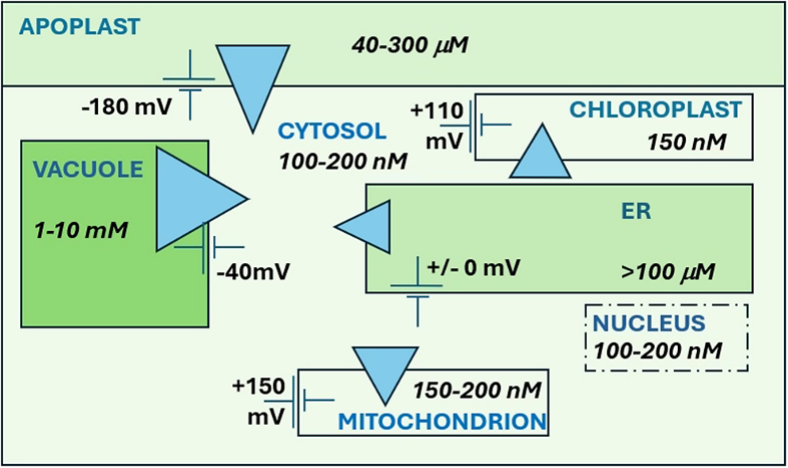
Ca^2+^ concentrations, gradients and membrane potentials in a typical plant cell. Blue triangles represent the magnitude and direction of the electrochemical potential gradient (ΔμCa^2+^).

The apoplast represents the primary source of Ca^2+^ entering a plant cell with variable apoplastic [Ca^2+^] reported from a number of studies (Figure 2; e.g. Felle & Hanstein, 2007; Conn et al., 2011; Stael et al., 2012). This, coupled with the large negative PM membrane potential (Vm) produces a large inward-directed electrochemical potential gradient (ΔμCa^2+^). Ca^2+^ entry across the plasma membrane occurs primarily via Ca^2+^-permeable channels, including cyclic nucleotide-gated channels (CNGCs), glutamate receptors (GLRs), and mechanosensitive channels (MSLs, MCas and OSCAs) (Basu & Haswell, 2017; Brownlee & Wheeler, 2023; Demidchick et al., 2018; Edel et al., 2017; Guichard et al., 2022; Jiang & Ding, 2023; Tian et al., 2020; Yoshimura et al., 2021). Nucleotide-binding leucine-rich repeat receptors (NLRs) mediate immune responses and cell death in response to pathogens. Two plant NLRs (N REQUIREMENT GENE 1 (NRG1) and ZAR1) have also been shown to form Ca^2+^ channels in *Arabidopsis* involved in Ca^2+^-mediated resistance to pathogen attack (Bi et al., 2021; Jacob et al., 2021).

Ca^2+^ entry channels can be activated in highly specific manners by a very wide range of biotic and abiotic stimuli and can be subject to multiple forms of regulation, including voltage-dependent activation and inactivation (Hille, 1978). For, example, in root hairs, CNGCs, including CNGC5, CNGC6, CNGC9 and CNGC14 play important roles in apically localised Ca^2+^ signalling (Tan et al., 2020; Tian et al., 2020). Notably, CNGC14 is inhibited by elevated [Ca^2+^_cyt_] via calmodulin (Zeb et al., 2020). In pollen tubes the Ca^2+^- permeable CNGC18/CNGC8 heterotetramer is preferentially localized at the growing tip and becomes active through interaction with calmodulin (CaM2) at low [Ca^2+^_cyt_], leading to increased Ca^2+^ influx (Frietsch et al., 2007; Gao et al., 2014; Pan et al., 2019; Tian et al., 2020). GLRs also contribute significantly to the shaping of the pollen tube Ca^2+^_cyt_ gradient (Michard et al., 2017; Tian et al., 2020) and regulation of their activity and localization involves CORNICHON homologues (AtCNIHs) (Wudick et al., 2018). In stomatal guard cells, three classes of channels are involved in the closure response to external stimuli and are differentially regulated in response to external cues. The kinase BIK1 activates OSCA1.3 in response to bacterial flagellin (flg22) (Thor et al., 2020). Abscisic acid (ABA) was recently shown to activate CNGC channels via the Ca^2+^-independent kinase OST1 (Yang et al., 2024). GLR channels, activated by external L-methionine were also shown to be involved in stomatal Ca^2+^ signalling, involving further Ca^2+^ channel activation via reactive oxygen (ROS) production (Kong et al., 2016). There are also numerous reports of depolarization- and hyperpolarization-activated Ca^2+^ channels in plants for which detailed electrophysiological information is available (Demidchick et al., 2018). Genes encoding depolarization-activated Ca^2+^ channels have only been identified in animals and those encoding hyperpolarization currents in guard cells or root hairs remain unidentified.

While Ca^2+^ entry across the PM may initiate elevation of [Ca^2+^_cyt_] during signalling events, there is good evidence that in many cases Ca^2+^ release from intracellular stores may account for the bulk of Ca^2+^ entering the cytosol:

#### 2.2.2. Channel-mediated Vacuolar Ca^2+^ release

The vacuole may occupy more than 90% of the total cell volume in a typical plant cell. While vacuolar free [Ca^2+^] ([Ca^2+^_vac_]) can vary substantially (Pottosin & Schonknecht, 2007), microelectrode measurements indicate [Ca^2+^_vac_] in the low mM range (Felle, 1988). This, coupled with a cytosol-negative membrane potential (Dindas et al., 2021) generates a large (ΔμCa^2+^) directed into the cytosol (Figure 2). Despite the obvious potential to represent the largest releasable Ca^2+^ store, the role of vacuolar Ca^2+^ release during signalling remains unclear. Direct involvement of the Ca^2+^-permeable slow vacuolar (SV) channel TPC1 (Peiter et al., 2005) as a major pathway for vacuolar Ca^2+^ release into the cytosol (Hedrich & Neher, 1987; Ward & Schroeder, 1994) was questioned following the finding that *Arabidopsis Attpc1* mutants did not show any differences from wild type in stomatal [Ca^2+^_cyt_] elevations in response to external ABA or methyl jasmonate (Islam et al., 2010). *Attpc1* mutants were able to close their stomata normally in response to ABA but showed either no response (Peiter et al., 2005) or a reduced response to increased external Ca^2+^ (Islam et al., 2010). Moreover, long-distance salinity- or wounding-induced root-shoot Ca^2+^ waves were respectively significantly slower or abolished in *Attpc1* mutants (Choi et al., 2014; Kiep et al., 2015).

Mechanosensitive tonoplast-localized Ca^2+^-permeable (PIEZO) channels have been shown to be involved in the transduction of mechanical signals in *Arabidopsis* root columnellar cells (Mousavi et al., 2021). Chimaeras of AtPIEZO with mouse mPIEZO generated non-selective mechanosensitive currents in HEK cells. PIEZO channels are therefore potentially involved in mechanosensitive Ca^2+^ release from the vacuole. PIEZO channels were also shown to contribute to Ca^2+^_cyt_ oscillations in the moss *Physcomitrium* and to be an important factor regulating vacuolar morphology (Radin et al., 2021).

**Table 1:** Examples of identified endomembrane transporters with demonstrated roles in modulation of Ca^2+^_cyt_ or organellar Ca^2+^.

Intracellular sources and sinks with demonstrated roles in regulating Ca^2+^_cyt_				
Compartment membrane	Transporters	Ca^2+^_cyt_ impact	Physiological process	References
Tonoplast	PIIB ATPase PCA1	↓	*Physcomitrium* salinity/osmotic signalling	Qudeimat et al., 2008
	TPC1?	↑ ↑	*Arabidopsis* long-distance signalling. Stomatal closure in response to external Ca^2+^ and ROS.	Cho et al., 2012; Islam et al., 2010; Kiep et al., 2015; Peiter et al., 2005
	AtACA4, ACA11	↓	Arabidopsis response to flg22 and maintenance of resting Ca^2+^_cyt_.	Frei et al., 2012; Hilleary et al., 2020
	AtPIEZO (AtPZO1) PpPIEZO	↑ ↑	Mechanotransduction in root cells. Ca^2+^_cyt_ oscillations in *Physcomitrium*	Mousavi et al., 2021 Radin et al., 2021
	AtCAX2	↓	Flooding and hypoxia Ca^2+^_cyt_ response modulation	Bakshi et al., 2023
	AtCCX2	↕?	Salt and osmotic responses	Corso et al., 2018
ER	AtECA1 AtACA1,2,7 NbCA1 AtCCX2	↓ ↓ ↓	Asymmetric Ca^2+^_cyt_ elevation in root bending and hydrotropic response. Arabidopsis response to flg and maintenance of resting Ca^2+^_cyt_. Responses to cryptogenic. Regulation of ER-cytosol Ca^2+^ exchange.	Shkolnik et al., 2018 Ishka et al., 2021 Corso et al., 2018
Intracellular organelle-specific Ca^2+^_cyt_ elevations				
Compartment membrane	Transporters	Organelle Ca^2+^ impact	Physiological process	References
Mitochondria	AtMCU1–3 AtMICU	↑ ↓	A major route for fast mitochondrial Ca^2+^ uptake. Required for jasmonic acid signalling and thigmomorphogenesis Modulates mitochondrial Ca^2+^ uptake by MCU and shapes mitochondrial Ca^2+^ signatures.	Ruberti et al., 2022; Teardo et al., 2017 Wagner et al., 2015
Chloroplast	AtMCU AtBICAT2 AtBICAT1	↑ ↑ ↓	Stromal Ca^2+^ accumulation in response to hyperosmotic shock. Imports Ca^2+^ across the chloroplast envelope. Underlies stromal Ca^2+^ increase in response to light–dark transition Transports Ca^2+^ into the thylakoid lumen. Modulates BICAT2-mediated stromal Ca^2+^ increase	Teardo et al., 2019 Frank et al., 2019
Nucleus	MtCNGC15/MtDMI1	↑	Nuclear envelope-ER localized channels coordinately mediate nuclear Ca^2+^_cyt_ oscillations in response to root nodulation factors	Liu et al., 2022 Charpentier et al., 2016

#### 2.2.3. Endoplasmic reticulum (ER)

While the Vm across the ER membrane has not been directly measured in plants, in animals ER Vm is clamped to near zero by high K^+^ conductance and equimolar [K^+^] on both sides of the ER membrane (Lam & Galione, 2013). However, an ER free [Ca^2+^] ([Ca^2+^_ER_]) >100 μM (Daverkausen-Fischer & Pröls, 2022) establishes a large cytosol−directed ΔμCa^2^ (Figure 2). While most studies of [Ca^2+^_ER_] changes in response to various stimuli do not report calibrated values in plants, lower affinity variants of GECI reporters (e.g. ER-GCAMP6) have been used successfully to report [Ca^2+^_ER_] changes (Resentini et al., 2021a). In animals, the Ca^2+^ release pathway from the ER is very well studied with both Inositol 1,4,5 trisphosphate (InsP_3_) and ryanodine receptors acting as the major pathways for Ca^2+^ release (Katona et al., 2022; Lam & Galione, 2013). However, while there are reports of InsP_3_-induced Ca^2+^ release in plant cells (e.g. Manzoor et al., 2012; Muir & Sanders, 1996), vascular plants do not possess canonical InsP_3_ or ryanodine receptors (Edel et al., 2017; Verret et al., 2010) and there is no characterized mechanism for facilitating Ca^2+^ release directly from the ER into the cytosol in plants. A clear exception, however, comes from work with root nodulation and mycorrhizal symbioses responses (Lam et al., 2024). Distinct from plasma membrane channel-mediated increases in [Ca^2+^_cyt_], mycorrhizal (Myc) factors from arbuscular mycorrhizal fungi and nodulation (Nod) factors from rhizobia can give rise to Ca^2+^ elevations localized to the nucleus (Charpentier et al., 2016; Lam et al., 2024; Oldroyd, 2013). In the legumes of *Medicago trunculata* or *Lotus japonicus*, Nod factor perception by LysM-type plasma membrane receptors is conveyed to the ER-derived nuclear envelope through a cytosolic mevalonate pathway (Venkateshwaran et al., 2015). Interaction between CNGC15 and the CASTOR/POLLUX/DMI1 channels on the inner nuclear envelope membrane underlies the release of Ca^2+^ into the nucleoplasm. While DMI1 has been proposed to behave as a K^+^ channel, more recent evidence suggests that both CNGC15 and DMI1 are Ca^2+^ channels (Kim et al., 2019).

While molecular evidence for specific pathways underlying ER-mediated Ca^2+^ release into the cytosol is lacking, there is clear physiological evidence for a role for ER Ca^2+^ release in Ca^2+^_cyt_ signalling. The mechanisms of trap closure by the Venus flytrap involve an initial depolarization of the trap lobe cell PM following mechanical stimulation of trap lobe hair cells. This involves an initial influx of Ca^2+^, most likely through GLR3.6 channels (Scherzer et al., 2022). Based on the effects of the ER Ca^2+^-ATPase inhibitor cyclopiazonic acid (CPA), which increased resting [Ca^2+^_cyt_] but decreased the amplitude of the transient Ca^2+^ elevation required for the trap closure response, Scherzer et al. (2022) deduced that an initial [Ca^2+^_cyt_] elevation associated with Ca^2+^ influx through GLR channels was augmented substantially by Ca^2+^ release from the ER. A similar role for ER Ca^2+^ release in elevation of [Ca^2+^_cyt_] has recently been indicated by Huang et al. (2023), who monitored Ca^2+^_cyt_ in *Arabidopsis* guard cells expressing a light-activated H^+^-permeable channelrhodopsin (HcKCR2) to generate H^+^-induced [Ca^2+^_cyt_] elevations. Pharmacological treatment with cyclopiazonic acid (CPA) or low apoplastic Ca^2+^ (EGTA) indicated a role in ER Ca^2+^ release. Moreover, repetitive stimulation led to a stepwise reduction of the Ca^2+^_cyt_ signals, indicating that ER Ca^2+^ stores could be depleted if Ca^2+^ release exceeded the recharging ability of ER-localized ATPases (see below). More recently, Huang et al. (2024) used the Ca^2+^-permeable, blue light-activated optogenetic probe channelrhodopsin ChR2-XXM2.0 to elicit pulses of Ca^2+^ influx across the PM of stomatal guard cells. This study showed that Ca^2+^ influx gave rise to Ca^2+^_cyt_ elevations, which were also associated with incremental closure of the stomata. CPA prevented the ChR-induced transient Ca^2+^_cyt_ elevation, presumably by inhibiting the loading of Ca^2+^_cyt_ into ER stores and provided evidence for ER store depletion with repetitive (every 6 min) Ca^2+^ release events.

### 2.3. *Transporters maintaining Ca*^*2+*^_*cyt*_ *homeostasis*

A combination of empirical and modelling approaches has provided evidence for the recovery phase of Ca^2+^ signals in determining the nature of downstream signalling responses (Lenzoni et al., 2018). The rate of recovery of Ca^2+^_cyt_ following elevation, and the set point for resting [Ca^2+^_cyt_] is largely determined by the affinity and regulated activity of Ca^2+^ extrusion systems on the PM and endomembranes (Table1). Two main classes of Ca^2+^-ATPases remove Ca^2+^ from the cytosol: Type PIIA (ER-type, ECA) located mainly, but not restricted to endomembranes, unlike animal ECA ATPases, and Type IIB, autoinhibited ATPases (ACA), which can be located at both PM and endomembranes (vacuole, ER, Golgi) (see Costa et al., 2023 for a recent review). ATPases exchange 1 Ca^2+^ for 1 or 2 H^+^, therefore utilising both the energy of ATP hydrolysis and the H^+^ electrochemical potential gradient. *Arabidopsis* AtACA8 comprises 10 transmembrane domains and a cytoplasmic head with nucleotide binding and CaM binding domains. Other regulatory mechanisms include phosphorylation (e.g. via CPK1/16, CIPK9/14, CBL1, CaM, CML36) and differences in ACA regulatory sequences likely reflect differential regulation (Costa et al., 2023). To date, only one ECA regulatory protein (MIZ1) has been identified (Yamazaki et al., 2012).

ATPases show diverse patterns of localization that are both cell-type and species-dependent (Costa et al., 2023). For example, *Arabidopsis* possesses 4 Type IIA ECAs and 10 Type IIB ACAs, 5 of which are located at the PM. ACA9 is expressed in pollen tubes while ACA8 and ACA10 are expressed in vegetative cells. ACA12 and ACA13 13 are preferentially expressed under biotic or abiotic stress. Both type IIA ECA and IIB ACA are found in the ER membrane. Two type II ACAs (ACA4 and ACA11) are localized to the vacuole. ATPases and other transporters potentially serve two roles. Firstly, to restore and maintain resting [Ca^2+^_cyt_] and secondly to charge up Ca^2+^ stores involved in Ca^2+^ release during signalling.

#### 2.3.1. Plasma membrane transporters

The roles of different Ca^2+^-ATPases have been inferred from both genetic and inhibitor studies (Costa et al., 2023; Demidchick et al., 2018). Mutant studies have revealed significant redundancy and have indicated important roles for localization in determining function (Costa et al., 2023; Resentini et al., 2021a; Resentini et al., 2021b). Examples include impaired pathogen defence responses and attenuated Ca^2+^_cyt_ signals of PM-localized *Ataca8/10* double knockout mutants in response to flg22 (Frei dit Frey et al., 2012). Behera et al. (2018) showed that resting Ca^2+^_cyt_ was unchanged in *aca8/10* double mutants, which also showed decreased Ca^2+^_cyt_ signal amplitude and delayed recovery in response to external ATP compared with wild-type plants. This response was considered to reflect a degree of acclimation via modified expression of other transporters. By monitoring both Ca^2+^_cyt_ and pH these workers also demonstrated that Ca^2+^ and pH fluxes were tightly linked.

#### 2.3.2. Tonoplast transporters

In contrast to mutants with disabled PM ATPases *Ataca4/11* double knockout mutants of vacuolar Ca^2+^-ATPases have elevated baseline [Ca^2+^_cyt_], elevated Ca^2+^_cyt_ response to CO_2_ and enhanced defence responses (Hilleary et al., 2020). By imaging [Ca^2+^_cyt_] with a YC-Nano65 sensor at both whole organ and sub-cellular scales, the flg22 response was found to be homogeneous across cells. Mis-localization of PM ACA8 suppressed the *aca4/11* phenotype, despite not having the same regulatory elements as ACA4/11. Perhaps surprisingly, while *aca4/11* mutants showed a significantly higher [Ca^2+^_cyt_] signal in response to flg22, [Ca^2+^_cyt_] returned to basal levels in a similar time frame as wild-type plants, suggesting the involvement of other efflux systems in re-establishing baseline [Ca^2+^_cyt_]. In contrast, knockout of the *Physcomitrium* tonoplast Ca^2+^-ATPase *PCA1* gave higher Ca^2+^_cyt_ transients in response to high NaCl, which were of longer duration than wild type (Qudeimat et al., 2008). These studies also indicated that vacuolar Ca^2+^-ATPases act very quickly to modulate the amplitude of the Ca^2+^_cyt_ signal.

CAX transporters belong to the multigene family of cation/H^+^ exchangers (Demidchick et al., 2018, Pittman & Hirschi, 2016). Plant CAX transporters have a lower affinity for Ca^2+^ than Ca^2+^-ATPases and transport H^+^ and Ca^2+^ in a 3:1 ratio (Demidchick et al., 2018; Dindas et al., 2021). *Arabidopsis* possesses 6 *CAX* genes (*AtCAX1–6*) and 5 further Ca^2+^/cation antiporters that behave as K^+^-dependent Na^+^/Ca^2+^ exchangers (Manohar et al., 2011; Maser et al., 2001, Shigaki et al., 2006). CAX transporters possess an N-terminal autoinhibitory domain and transport specificity is controlled by a 9 amino acid region between TM1 and TM2. Activity depends on ΔμH^+^ across the tonoplast, the degree of phosphorylation and interaction of regulatory proteins with the N-terminal region (Demidchick et al., 2018; Matthew et al., 2024; Pittman & Hirchi, 2016; Wang et al., 2024).

Despite their lower affinity, physiological and molecular studies indicate that CAX transporters play a pivotal role in the regulation of Ca^2+^_cyt_ dynamics. They also play important roles in the regulation of cytosolic and apoplast pH (Cho et al., 2012). *Arabidopsis det3* V-type H^+^-ATPase mutants (Allen et al., 2000) had altered Ca^2+^ dynamics in response to increased apoplastic Ca^2+^ and ROS, displaying sustained stomatal guard cell Ca^2+^_cyt_ elevations rather than oscillations normally associated with these stimuli, likely reflecting defective Ca^2+^/H^+^ regulation *via* Ca^2+^/H^+^ transporters. However, *det3* mutants did show a normal pattern of Ca^2+^_cyt_ oscillations in response to ABA. Electrophysiological manipulation of tonoplast Vm combined with Ca^2+^_cyt_ imaging in *Arabidopsis* root hairs has provided further evidence for tonoplast Ca^2+^/H^+^ exchange in the regulation of [Ca^2+^_cyt_] (Dindas et al., 2021). Depolarizing the tonoplast (i.e. rendering the cytosolic side more positive) elevated [Ca^2+^_cyt_] and reduced [H^+^]_cyt_. This can be interpreted as reduced ΔμH^+^ leading to reduced Ca^2+^ uptake into the vacuole in exchange for H^+^. Hyperpolarizing the tonoplast produced the opposite effect. Two recent reports provide further direct evidence for the roles of CAX in the maintenance of Ca^2+^_cyt_ homeostasis. Bakshi et al. (2023) showed transcripts of *CAX2* and *ACA1* were rapidly upregulated in *Arabidopsis* plants subject to flooding or hypoxia. Moreover, *cax2* knockout mutants showed larger and more sustained Ca^2+^_cyt_ signals and enhanced survival in response to flooding. In a separate study (Conn et al., 2011) *Arabidopsis cax1/3* double mutants had reduced overall mesophyll Ca^2+^ contents and apolastic free Ca^2+^ that was 3-fold higher than wild-type plants. This was indicative of compensatory increased PM ATPase activity in response to reduced tonoplast Ca^2+^ transport.

#### 2.3.3. ER transporters

There are numerous reports of disruption of ER Ca^2+^-ATPases leading to altered Ca^2+^_cyt_ signalling and downstream responses (Costa et al., 2023). Analysis of Ca^2+^_ER_ dynamics in pollen tubes expressing ER-localized yellow cameleon 3.6 Ca^2+^ sensor showed that CPA triggered growth arrest and a decrease in [Ca^2+^_ER_] (Iwano et al., 2009). CPA also reduced the tip-focused Ca^2+^_cyt_ oscillations in the growing pollen tube tip and caused [Ca^2+^_cyt_] to elevate in sub-tip regions indicating a key role for ER Ca^2+^-ATPase in regulating the tip-focused [Ca^2+^_cyt_] gradient. More specifically, *Arabidopsis* triple mutants *aca1/2/7* of ER-localized Ca^2+^-ATPase show higher Ca^2+^_cyt_ response to flg22 or blue light, higher resting [Ca^2+^_cyt_] and associated changes in downstream responses (Ishka et al., 2021). Triple *aca1/2/7* mutants also had slower recovery of [Ca^2+^_cyt_] to resting levels following repeated cycles of elevated CO_2_ as well as altered stomatal conductance (Jezek et al., 2021). Increases in [Ca^2+^_cyt_] were also progressively reduced in mutants in response to successive CO_2_ cycles, indicating that ACA-mediated recovery of the ER Ca^2+^ store was required for response to repeated stimuli. A further role for Ca^2+^-ATPase in shaping Ca^2+^ signatures comes from studies of nod factor signalling in *Medicago.* Silencing the nuclear envelope-localized Ca^2+^-ATPase MCA8 blocked nod factor-induced nuclear Ca^2+^ oscillations (Capoen et al., 2011).

A role for an ER-localised cation/Ca^2+^ exchanger (CCX) in the regulation of both [Ca^2+^_ER_] and [Ca^2+^_cyt_] in *Arabidopsis* has also been demonstrated (Corso et al., 2018). Surprisingly, knockout of *AtCCX* resulted in decreased [Ca^2+^_cyt_] and increased [Ca^2+^_ER_] under salt and osmotic stress conditions. The underlying mechanism and role in cytosol-ER Ca^2+^ exchange have yet to be fully elucidated.

## 3. Mitochondrial and chloroplast Ca^2+^ transport

In animal cells, mitochondrial Ca^2+^ uptake is critical for control of energy metabolism and mitochondria play a fundamental role in shaping spatio-temporal Ca^2+^_cyt_ increases. This occurs primarily via InsP_3_-induced release of Ca^2+^ from ER stores during Ca^2+^_cyt_ wave propagation and reuptake of Ca^2+^ by mitochondrial Ca^2+^ uniporters (MCUs) at specific locations where mitochondria make close contact with the ER (ER-mitochondrial contacts, ERMCs) (Katona et al., 2022; Lee et al., 2018). Mitochondria maintain a large inside-negative Vm across the inner mitochondrial membrane (Zorova et al., 2018) resulting in an inwardly-directed ΔμCa^2+^, given mitochondrial matrix Ca^2+^ [Ca^2+^_mit_] of 100–200 nM (Finkel et al., 2015) (Figure 2). While there is little or no evidence for functional ERMCs in plant cells, recent work has shown that MCU proteins play a key role in Ca^2+^ uptake. *Arabidopsis* possesses 6 MCU homologues (Teardo et al., 2017) and Ruberti et al. (2022) demonstrated in vitro Ca^2+^ transport activity by *Arabidopsis* MCU and defective mitochondrial Ca^2+^ uptake in a *mcu1/2/3* triple knockout mutant. However, this study also showed that Ca^2+^_cyt_ dynamics were unaffected in the triple mutant, indicating that mitochondria played a minimal role in Ca^2+^_cyt_ homeostasis. A homologue of the animal regulatory MCU-associated MICU proteins has been implicated in the regulation of MCU-mediated Ca^2+^ uptake (Wagner et al., 2015). *Arabidopsis mice* mutants showed higher and faster mitochondrial (Ca^2+^_mit_) elevations in response to auxin and ATP while Ca^2+^_cyt_ remained unchanged. Thus, similar to animal cells, by modulating plant mitochondrial Ca^2+^ uptake MICU shapes mitochondrial Ca^2+^ signatures and helps to maintain mitochondrial Ca^2+^ homeostasis.

Similar to mitochondria, chloroplasts have a large inside negative membrane potential (Svabo & Spetea, 2017) and a stromal [Ca^2+^] ([Ca^2+^_strom_]) of 50–200 nM (Frank et al., 2019; Hochmal et al., 2015), maintained by active transporters such as the thylakoid membrane-localized Ca^2+^/H^+^ exchanger CCHA1 (Wang et al., 2016). A chloroplast-localized homologue of mitochondrial MCU transporters (cMCU) has also been identified and shown to mediate chloroplast Ca^2+^ uptake (Teardo et al., 2019). By using targeted aequorin reporters they showed that cMCU was required for stress-specific Ca^2+^_strom_ signatures. The dynamic nature of chloroplast Ca^2+^ signalling is further illustrated by responses of Ca^2+^_strom_ to high light and temperature. By targeting YC3.6 to the cytosol, chloroplast and mitochondria in the green alga *Chlamydomonas*, Pivato et al. (2023) monitored elevated Ca^2+^ specific to the chloroplast, which correlated with H_2_O_2_ production and was dependent on functional cryptochrome. Similarly, Flori et al. (2024) demonstrated sustained Ca^2+^_strom_ elevations in response to high light or H_2_O_2_ treatments that were independent of changes in [Ca^2+^_cyt_] and accompanied chloroplast H_2_O_2_ accumulation in high light. A further class of chloroplast Ca^2+^ transporter, BICAT proteins, are involved in the elevation of Ca^2+^_strom_ on transfer from light to dark (Frank et al., 2019). Knockout mutations of *BICAT1*, which is located on the chloroplast envelope reduced the dark-induced Ca^2+^_strom_ signal, monitored with chloroplast-targeted aequorin. In contrast, knockout mutation of *BICAT2*, which transports Ca^2+^ into the thylakoid lumen increased the light-dark Ca^2+^_strom_ signal and produced severe defects in chloroplast morphology.

## 4. Insights from modelling studies

A number of modelling studies have provided insights into the interactions between signalling and homeostatic components of the plant Ca^2+^ signalling machinery. Bose et al. (2011) used a 4-component model, comprising 2 Ca^2+^-permeable channels on the PM and endomembrane, together with 2 efflux systems – a PM Ca^2+^-ATPase and an endomembrane Ca^2+^/H^+^ exchanger. They also factored in the ROS sensitivity of the endomembrane Ca^2+^ channel and the buffering capacity of the cytosol. The model predicted that specific Ca^2+^ signatures could be achieved by modifying the activities of ATPase and Ca^2+^/H^+^ exchangers. Dindas et al. (2021) combined electrophysiological manipulation of vacuolar Vm, Ca^2+^_cyt_ monitoring and modelling to demonstrate the role of a voltage-dependent vacoular Ca^2+^ homeostat involving tonoplast Ca^2+^/H^+^ exchange and vacuolar electrical excitability providing a clear demonstration that Ca^2+^ fluxes across the tonoplast are important in regulating Ca^2+^_cyt_.

The “*On guard*” model (Jezek et al., 2021) presents arguably the most comprehensive analysis of transport and essential metabolism in predicting stomatal signalling patterns and behaviour. The model considers Ca^2+^- and H^+^-ATPases, along with cation and anion channel activities shown to be associated with stomatal responses to ABA or CO_2_. The model accurately simulated elevated [Ca^2+^_cyt_] and oscillations resulting from cyclic Ca^2+^ influx across the PM, promoting much larger Ca^2+^-induced Ca^2+^ release (CICR) from endomembrane stores. Modelling also predicted, and experiments verified, a delay in Ca^2+^ cycling that was enhanced in ER and tonoplast Ca^2+^-ATPase mutants, identifying both endomembrane Ca^2+^-ATPases and Ca^2+^ channels as important targets for the stomatal closure response to high CO_2_.

## 5. Outlook

There has been substantial progress in understanding the essential components of the plant Ca^2+^ homeostatic machinery and how these contribute to the shaping of Ca^2+^ signals in response to a wide range of stimuli in different plant, tissue and cell types. This progress has been facilitated largely by advances in molecular characterization of key transporters, along with the development of targeted genetically encoded indicators with differing affinities suited to imaging Ca^2+^ in the cytosol and other cellular compartments, along with increasingly refined modelling simulations. Technological advances in microscopy from sub-cellular to whole plant imaging have further enabled spatio-temporal Ca^2+^ signalling patterns to be analysed in far greater depth than previously possible. Discoveries enabled through studies of a wider range of organisms, such as the recently described MID1-COMPLEMENTING ACTIVITY (MCA) PM Ca^2+^-permeable channel with a role in the regulation of cell proliferation in the liverwort *Marchantia polymorpha* (Iwano et al., 2025) will provide further insights into the roles and evolution and of plant Ca^2+^ signalling components.

Despite these advances, there is still a need to further define the roles of endomembrane stores as sources and/or sinks for Ca^2+^. There has been substantial progress in understanding how both PM and endomembrane Ca^2+^-ATPases and CAX transporters can shape different Ca^2+^ signatures, though with still much to discover. While CNGCs have been identified as the major Ca^2+^ release pathway from the nuclear envelope underlying nuclear Ca^2+^ oscillations during symbiotic signalling, our more general understanding of the roles of endomembrane Ca^2+^ release mechanisms is less advanced. How is an initial Ca^2+^ influx across the PM augmented and amplified by the release of Ca^2+^ from internal stores? Of particular importance is the ongoing need to identify ER Ca^2+^ release mechanisms in the absence of molecular homologues of InsP_3_ receptors that are widespread in animal cells and play a central role in Ca^2+^ signalling. There is also a need to further understand the interactions between different endomembrane compartments. In animals, interactions between ER and mitochondria are pivotal in defining spatiotemporal Ca^2+^ signalling. To date, no similar evidence exists for plant cells. Are there similar direct interactions between the ER and mitochondria or chloroplasts in plants? The vacuole represents the largest potential Ca^2+^ stored in most plant cells. However, while tonoplast Ca^2+^ transporters have been shown to play a role in Ca^2+^_cyt_ homeostasis, apart from the recently discovered tonoplast PIEZO channels (Mousavi et al., 2021; Radin et al., 2021) there is no fully characterized channel-mediated mechanism for vacuolar Ca^2+^ release associated with specific Ca^2+^ signalling events.

Finally, in animals, store-operated Ca^2+^ entry (SOCE) is an important mechanism for recharging ER stores during repetitive Ca^2+^ signalling and is a key component in shaping Ca^2+^ signatures. However, the molecular machinery for SOCE – STIM proteins that sense [Ca^2+^_ER_] and ORAI channels in the PM that allow Ca^2+^ entry in close proximity to ER-PM contact points (Lunz et al., 2019; Wang et al., 2014) are absent in embryophytes, though ORAI proteins are present in the green lineage as far as gymnosperms (Edel et al., 2017). This begs the question of whether SOCE exists, at least in embryophytes.

## Data Availability

This review article does not rely on original data or resources.
